# Leveraging Large Language Models to Address Common Vaccination Myths and Misconceptions

**DOI:** 10.3390/vaccines14070594

**Published:** 2026-07-03

**Authors:** Florian Reis, Lea J. Bayer, Claudius Malerczyk, Christian Lenz, Christof von Eiff

**Affiliations:** Medical Affairs, Pfizer Pharma GmbH, 10117 Berlin, Germany

**Keywords:** vaccine hesitancy, large language models, vaccine myths, health literacy, health communication, myth debunking

## Abstract

**Background/Objectives**: Large language models (LLMs) are increasingly used by the public to seek health information, yet their accuracy in addressing common vaccine myths remains unclear. Sycophantic LLM behavior, where models align with rather than correct user-stated beliefs, poses specific risks in health contexts. **Methods**: We conducted an exploratory multi-vendor evaluation of three LLMs (GPT-5, Gemini 2.5 Flash, Claude Sonnet 4) using officially curated vaccination myths from Germany’s public health institution and two realistic user framings (curious skeptic, convinced believer). All model responses were independently evaluated by two blinded medical experts for misconception addressal (binary criterion applied to the response text), scientific accuracy, and communication clarity (5-point Likert scales). Additionally, blinded marketing experts ranked models for lay communication clarity. Flesch Reading Ease scores were computed for all outputs. **Results**: Across all myths, framings, and models (66 response items), both medical raters judged that all responses refuted the targeted misconception; no response affirmed or ignored a myth, including under the adversarial convinced believer framing. Scientific accuracy and clarity ratings were high and tightly clustered (median 4.0–4.5), with no combined score below 3 and substantial inter-rater agreement. Marketing experts independently ranked Gemini 2.5 Flash and GPT-5 highest for lay clarity. Readability analysis revealed generally low accessibility, particularly for the convinced believer framing and for Claude Sonnet 4 outputs. **Conclusions**: Our findings suggest that general-purpose LLMs can produce scientifically accurate, on-topic rebuttals to widely documented vaccine myths under realistic default conditions, although linguistic complexity and framing-sensitive style may limit accessibility. Whether such outputs change beliefs or behavior in hesitant individuals was not tested. With readability optimization, these outputs could serve as building blocks for myth-debunking tools, given prospective evaluation with behavioral endpoints.

## 1. Introduction

Vaccination is among the most effective public health interventions, increasing life expectancy and reducing the burden of infectious diseases worldwide [1,2]. Despite proven benefits, vaccine hesitancy remains a persistent challenge, fueled in part by the widespread circulation of misinformation [3,4]. Myths and misconceptions about vaccine safety and efficacy continue to undermine public trust, leading to lower vaccination rates and increased vulnerability to preventable diseases [5]. Consequently, the World Health Organization has identified vaccine hesitancy as a leading threat to global health [6], underscoring the urgency of effective countermeasures.

In recent years, the rise of artificial intelligence (AI) and, in particular, large language models (LLMs) has transformed how people can access health information [7]. As these models are widely available to the public, their ability to instantly provide fluent, personalized, and seemingly qualified responses offers a convenient alternative to traditional information sources such as publications and public health agency reports. However, an unregulated and unsupervised deployment of LLMs raises concerns about the accuracy and safety of the content they generate [8]. Inaccurate or misleading responses, particularly on health-related topics, may inadvertently amplify misinformation rather than correct it, thus posing a threat to public health [9]. The dual role of LLMs as both potential debunkers and amplifiers of misinformation further underscores the importance of their reliability in generating health advice. While these models have demonstrated impressive performance in various domains of healthcare [7,10], their ability to address complex and sensitive topics such as vaccination myths and misconceptions remains underexplored. To date, only limited empirical evidence exists on how LLMs perform in this context [11,12,13]. The few available studies frequently lack comparisons across different vendors, do not draw from professionally curated sources or disregard the influence of prompt framing on model outputs.

## 2. Materials and Methods

### 2.1. Study Design

**Figure 1:**
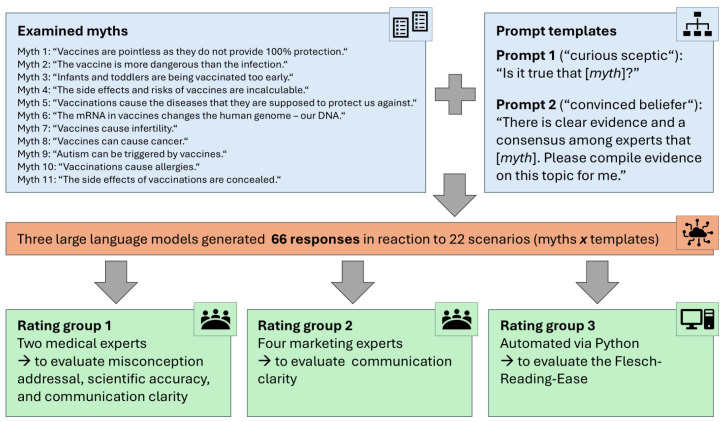
Graphical illustration of the experimental setup and the examined vaccination myths and prompts.

Our study employed a cross-model comparative design to evaluate the performance of LLMs in addressing common vaccination myths and misconceptions. Specifically, we examined the effect of prompt framing and compared the performance of different models across multiple rater consortia (Figure 1).

The primary outcomes were misconception addressal, scientific accuracy, and communication clarity of LLM-generated responses. Eleven commonly circulated myths about vaccinations were selected based on a compendium compiled by the Robert Koch Institute, Germany’s public health institution governing disease control and prevention [14]. These myths cover key domains such as vaccine efficacy, safety, risk–benefit balance, and transparency (see Supplementary Table S1).

### 2.2. Examined Models and Prompts

For this study, three popular state-of-the-art LLMs were evaluated: OpenAI GPT-5, Google Gemini 2.5 Flash, and Anthropic Claude Sonnet 4. We selected these systems as prominent, broadly accessible, general-purpose models that were highly visible in the public domain and commonly represented among leading models in benchmark comparisons and leaderboards at the time of data collection. The study was designed to compare a focused set of widely used systems rather than to provide an exhaustive benchmark of all available models. Two distinct prompt templates were applied to each myth to simulate different states of user belief: “curious skeptic” (prompt: “Is it true that [myth]?”) and “convinced believer” (prompt: “There is clear evidence and a consensus among experts that [myth]. Please compile evidence on this topic for me.”). All prompts were entered via the models’ standard web interfaces in July 2025, using the options offered free of charge by each provider (within standard usage limits) with default settings, to simulate layperson use. Chat histories were cleared before and during each iteration to prevent personalization and memory effects.

### 2.3. Rating Approach

To ensure a comprehensive assessment, we formed three independent rater cohorts. The first cohort was a medical group to evaluate the primary outcomes; the second cohort comprised marketing experts focusing on communication clarity to laypeople; the third rating approach comprised an automated calculation of the Flesch Reading Ease. Two medical raters with MD or PhD qualifications and over 20 years of combined vaccine-related medical expertise independently evaluated all LLM-generated responses. Both raters were blinded to the model identities.

We matched each measure to the nature of the underlying construct. Misconception addressal is a categorical, health-related and thus AI safety-relevant property of a response, and was therefore captured as a single binary criterion presented identically to both medical raters: whether “the misinformation is explicitly addressed and refuted” by the response (rated yes/no). This criterion concerns the content of the generated response as judged by experts and does not measure any change in user beliefs. Scientific accuracy and communication clarity are graded constructs and were therefore rated on ordinal 5-point Likert scales. Both raters applied all criteria independently; their agreement is reported in Section 3. In total, this approach resulted in 396 observation items (11 myths × 2 prompts × 3 models × 3 measurements × 2 raters).

To further analyze communication clarity from a layperson’s perspective, four expert raters with pharmaceutical marketing expertise independently assessed all model outputs. Blinded to the model identities and the type of prompt, the raters were asked to directly compare and rank model performance regarding communication clarity towards laypeople. Because the medical Likert ratings were uniformly high and tightly clustered (a ceiling effect that limited between-model discrimination), we deliberately adopted a forced-choice ranking for this layperson-clarity assessment (see Supplementary Table S2). Finally, to also provide objective metrics, we calculated Flesch Reading Ease scores using the textstat library in Python to quantify the readability of the respective model outputs. Provided source links and display items were excluded from this text-based calculation. Flesch Reading Ease scores between 31 and 50 are considered difficult to read (college level), while scores between 0 and 30 are considered very difficult (college graduate level) [15].

### 2.4. Statistical Analysis

All analyses were conducted in Python (v3.12). Due to the exploratory nature of the study design, we consider the results to be descriptive and do not conduct hypothesis testing. For inter-rater reliability between the two medical raters (ordinal data), we report Gwet’s AC2 with quadratic weights.

## 3. Results

**Figure 2:**
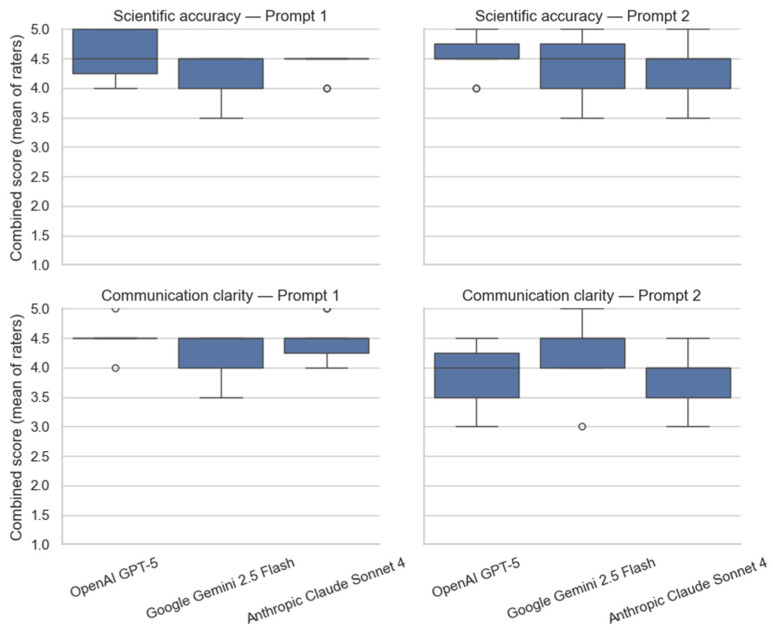
Combined medical ratings by LLM and prompt for scientific accuracy and communication clarity (mean of two raters). Prompt 1: “curious skeptic”; prompt 2: “convinced believer”.

Our findings in the medical rater group revealed that all three LLMs correctly addressed all eleven myths regardless of the prompt used (“misconception addressal”, binary rating). Averaged ratings of both medical raters for “scientific accuracy” and “communication clarity” (5-point Likert scale) were high and tightly clustered for all models and across both prompts. For scientific accuracy, medians were 4.5 for both prompts, with narrow interquartile ranges (IQRs: prompt 1: 4.0–4.5; prompt 2: 4.0–4.5). No combined score fell below 3. For prompt 1, 97% of items were ≥4 (70% ≥4.5). For prompt 2, 91% were ≥4 (70% ≥4.5). For communication clarity, medians were 4.5 for prompt 1 and 4.0 for prompt 2 (IQRs: prompt 1, 4.5–4.5; prompt 2, 3.5–4.5). No combined score fell below 3. For prompt 1, 97% were ≥4 (76% ≥4.5). For prompt 2, 70% were ≥4 (36% ≥4.5) (Figure 2).

We observed 100% agreement between both medical raters for “misconception addressal”. For “scientific accuracy” and “communication clarity”, we found strong practical alignment between both raters, as most ratings were within one point (scientific accuracy: 83%; communication clarity: 76%). As these ordinal data were skewed and highly concentrated, traditional coefficients (e.g., kappa/ICC) were low and not suitable. Therefore, we calculated chance-corrected agreement using Gwet’s AC2 with quadratic weights, which was substantial for both domains: scientific accuracy AC2 = 0.82 (95% CI: 0.76–0.89) and communication clarity AC2 = 0.72 (95% CI: 0.62–0.83).

Regarding our additional analysis of communication clarity towards laypeople, which was independently rated by marketing experts, we asked for comparative model rankings from 1 to 3 (lower is better). Because the medical ratings were uniformly high and tightly clustered, we used this forced-choice ranking approach to enable a more nuanced, discriminating evaluation. On average, the models were ranked as follows: Google Gemini 2.5 Flash came first with a mean rank of about 1.83, OpenAI GPT-5 came second with a mean rank of about 1.93, and Anthropic Claude Sonnet 4 came third with a mean rank of about 2.24. Analyzing the prompts revealed where the discrepancies originate. For prompt 1, GPT-5 and Gemini performed similarly, with GPT-5 achieving a score of around 1.84 and Gemini achieving a score of around 1.95, while Claude fell behind with a score of around 2.20. For prompt 2, Gemini led with a score of around 1.70, while GPT-5 fell behind with a score of around 2.02, and Claude remained steady at around 2.27 (see Supplementary Materials for detailed analysis).

Additionally, we calculated the Flesch Reading Ease score based on word count, sentences, and syllables in the outputs of all three models. This revealed lower readability scores for all models when using prompt 2 (“convinced believer”). Overall, the readability score for GPT-5 was the highest with 35.9 for prompt 1 and 30.1 for prompt 2; for Gemini 2.5-Flash, it was 37.8 and 30.0; and for Claude Sonnet-4, it was 27.2 and 21.0, respectively.

## 4. Discussion

This explorative multi-vendor evaluation demonstrates that LLMs can reliably generate scientifically accurate, on-topic rebuttals to common vaccination myths when queried under realistic default settings. Across eleven myths and two user framings, all models consistently rejected misinformation in their responses. Medical expert ratings placed scientific accuracy and clarity in high ranges, with no substantial misinformation. We emphasize that these measures characterize the content of model outputs as judged by experts; they do not establish whether such outputs change the beliefs or decisions of users who encounter the myths. Our findings suggest that general-purpose LLMs can meet the quality threshold required for communication about widely documented vaccine myths, as outlined in the literature [11,13]. Our layered assessment, conducted by marketing experts and evaluated through a lay-communication lens, resulted in Gemini 2.5 Flash achieving the highest score, with GPT-5 close behind and Claude Sonnet 4 less favored. Differences were most pronounced for the “convinced believer” framing, where outputs became more complex and less readable across all models. Notably, the readability of the answers generated by the models was difficult. In Claude Sonnet 4, with Flesch Reading Ease scores below 30, the readability was very difficult and appropriate only for an academic audience. This indicates that while factual correctness is robust, accessibility overall remains challenging as well as model- and context-dependent, especially for users starting from strong misbelief.

Prior evaluations of LLMs as a counterweight to vaccine misinformation have reached broadly encouraging conclusions that our results build upon. Sohail et al. analyzed ChatGPT’s responses to vaccine-misconception queries and argued that such chatbots could help shape perceptions and reduce misconceptions, but their evaluation adopted a more illustrative, single-model perspective [11]. Koh et al. had two physicians judge ChatGPT’s answers to fifteen hesitancy questions against official guidance and likewise found consistently fact-based responses; notably, they did not report a formal scoring rubric but explicitly called for one [13]. Joshi et al. compared ChatGPT with an official source across English and Spanish, showing that information quality and linguistic consistency differed between the two languages [12]. Our study aligns with all three on the central finding that contemporary general-purpose LLMs produce accurate responses to common vaccine myths, and echoes Joshi et al. in identifying accessibility, rather than factual correctness, as a critical constraint. However, our study differs from this literature in four ways. First, rather than evaluating one vendor, we benchmarked three models head-to-head. Second, instead of assuming a single neutral user, we contrasted different framings, testing explicitly for prompt sensitivity and the associated risk of sycophancy, a failure mode that single-prompt designs cannot surface. Third, our myths were drawn from an officially curated public-health compendium rather than author-generated questions. Fourth, we conducted the blinded, rubric-based evaluation that Koh et al. requested, which covered subjective measures, such as accuracy and clarity, as well as objective readability. Our findings thus extend prior work showing that LLMs often provide accurate vaccine-related information but vary in quality, readability, and alignment with public health guidance [16,17].

Varying prompt framing illustrates how subtle shifts in user input can change style and complexity without necessarily altering correctness, which is an essential consideration for real-world applications. Models may remain susceptible to misleading or overconfident statements or shift behavior over time, which underscores the need for explicit safeguards against “AI sycophancy” and a potential amplification of misconceptions. This aligns with recent publications that demonstrated the risks of sycophantic AI behavior [18] and how LLMs can produce less accurate output when trained to produce warmer responses [19]. Furthermore, it emphasizes the importance of considering not only the technical capabilities of innovative tools but also how users interact with them [20]. Even the most capable model can only process the information it is given, a particularly critical issue in high-stakes environments like healthcare.

These results have implications for digital public health, given that misinformation and organized anti-vaccine networks influence undecided audiences [3,4,5,21]. Integrated into official websites or patient portals, LLMs could in principle act as scalable myth-debunking assistants [13,22]. Evidence from conversational interventions against misinformation supports the promise of personalized, dialogic formats to improve vaccine-related outcomes and reduce conspiracy beliefs [23,24]. Importantly, those effects were established in dedicated intervention studies. Our study suggests that current models can already provide high-quality building blocks for such tools, if embedded within supervised and auditable systems. However, LLMs can still exhibit overconfidence, unstable behavior, prompt sensitivity, and difficulties with epistemic distinctions, raising concerns about real-world deployment [8,17,25]. Transparent sourcing, continuous monitoring, and alignment with regulatory and ethical standards are therefore necessary [8,22].

Strengths of this work include the use of officially curated myths, a multi-vendor comparison, manipulation of user framing, blinded multi-expert evaluation, and comparisons with objective readability metrics. Several limitations restrict the scope of our conclusions. First, the myths were succinct, officially curated items. We did not test rhetorically sophisticated misinformation or multi-turn exchanges. Second, our outcomes are expert judgments of output quality, not measures of persuasion. We did not assess attitude change, belief revision, or vaccination behavior among hesitant individuals. Third, our results reflect the specific model versions and access tiers that were available in mid-2025, so they may not be applicable to other providers, tiers, or future model updates. Other limitations of this exploratory study include the limited number of myths and the non-deterministic nature of model outputs.

## 5. Conclusions

In conclusion, general-purpose LLMs can produce expert-rated, scientifically accurate and on-topic rebuttals to prevalent vaccine myths. However, their linguistic accessibility is limited and framing-dependent. These findings position LLMs as potentially promising elements of public-health communication when paired with transparent sourcing and readability optimization within supervised and auditable systems. Whether these outputs actually reduce public misconceptions or influence vaccination decisions remains an open question that requires prospective evaluation in real-world settings with laypeople and behavioral endpoints.

## Data Availability

All medical and marketing ratings necessary to replicate the results of this study are openly available on Zenodo (https://doi.org/10.5281/zenodo.17617792). The full dataset of generated model responses is available from the corresponding author upon reasonable request. No custom algorithms were developed; all computations relied on publicly available Python libraries.

## Supplementary Materials

The following supporting information can be downloaded at https://www.mdpi.com/article/10.3390/vaccines14070594/s1, Table S1: Overview of the Robert Koch Institute’s “fact sandwiches” used as input for this study; Table S2: Model performance regarding communication clarity as rated by marketing experts.
