# Supplementary material for: Leveraging Large Language Models to Address Common Vaccination Myths and Misconceptions
Source: Vaccines (Basel). 2026 Jul 3;14(7):594. doi: 10.3390/vaccines14070594 (PMC13418006; doi:10.3390/vaccines14070594)
Supplement: Supplementary file 1 [file vaccines-14-00594-s001.zip › vaccines-4384640-supplementary.pdf]

Supplementary Materials

Leveraging large language models to address common vaccination myths and misconceptions

Table S1. Overview of the Robert Koch Institute's "fact sandwiches" used as input for this study*.

Topic	Myth	Fact
Efficacy	"Vaccines are pointless as they do not provide 100% protection."	"Vaccination is meaningful even though protection be 100 %."
Risk-benefit analysis	"The vaccine is more dangerous than the infection."	"The infection and its possible complications are significantly more dangerous than the recommended vaccine."
Childhood vaccination	"Infants and toddlers are being vaccinated too early."	"Vaccinating infants and toddlers is important for their health."
Safety	"The side effects and risks of vaccines are incalculable."	"The risks and side effects of vaccines are determined in clinical trials prior to their approval, and following approval they continue to be monitored worldwide."
Safety	"Vaccinations cause the diseases that they are supposed to protect us against."	"Vaccines cannot cause the disease they are meant to protect against."
Safety	"The mRNA in vaccines changes the human genome – our DNA."	"The mRNA in vaccines cannot be incorporated into the genome of our cells."
Safety	"Vaccines cause infertility."	"Vaccines have no effect upon fertility and are safe for those planning to have children."

Safety	“Vaccines can cause cancer.”	“Vaccines can protect against cancer.”
Safety	“The measles, mumps, and rubella vaccine can cause autism.”	“The measles, mumps, and rubella vaccination can be ruled out as a possible cause of autism.”
Safety	“Vaccinations cause allergies.”	“Vaccines do not cause allergies.”
Communication	“The side effects of vaccinations are concealed.”	“All known side effects of vaccinations are communicated via different channels.”

*Source of RKI Fact Sandwiches: https://www.rki.de/EN/Topics/Infectious-diseases/Immunisation/Information-material/Vaccination-myths/effectively-debunking-misinformation-node.html (accessed on July 1, 2025)

Table S2. Model performance regarding communication clarity as rated by marketing experts. Smaller mean ranks indicate better performance (scale: 1 = best, 2 = intermediate, 3 = worst).

**Model (Vendor)**	**Mean Rank (Standard Deviation)**	**Median**	**Absolute Count Rank 1**	**Relative Count Rank 1**
GPT-5 (OpenAI)	1.93 (0.084)	2.0	30	34.1%
Gemini 2.5 Flash (Google)	1.83 (0.087)	2.0	38	43.2%
Claude Sonnet 4 (Anthropic)	2.24 (0.086)	2.0	20	22.7%
